# Ecological Modulation of Soil Microbial Communities by Fertilization Regimes: Insights from Castor Bean Cake, Chemical Fertilizers, and Organic Fertilizer

**DOI:** 10.3390/microorganisms13122841

**Published:** 2025-12-14

**Authors:** Chongyang Hu, Yalijuan Wu, Zecheng Li, Zhiyong Wang, Fenglan Huang, Zhiquan Fan, Mu Peng

**Affiliations:** 1 Hubei Key Laboratory of Biological Resources Protection and Utilization, Hubei Minzu University, Enshi 445000, China; hcy1784364088@outlook.com (C.H.); 13039696187@163.com (Y.W.); 15545428880@163.com (Z.L.); wangzhiyong@hbmzu.edu.cn (Z.W.); 2021009@hbmzu.edu.cn (Z.F.); 2 College of Biological and Food Engineering, Hubei Minzu University, Enshi 445000, China; 3 College of Life Science and Food Engineering, Inner Mongolia Minzu University, Tongliao 028000, China; huangfenglan@imun.edu.cn

**Keywords:** soil microbial community, castor bean cake, community assembly, co-occurrence network

## Abstract

Fertilization plays a vital role in replenishing soil nutrients, shaping microbial community composition, and enhancing agricultural productivity. Castor bean cake (CBC) is a nitrogen- and carbon-rich by-product increasingly used as an organic amendment, yet its effects on soil microbiomes remain unclear. Here, we compared CBC with a compound chemical fertilizer (CF) and a manure-based organic fertilizer (OF) across dose gradients using 16S rRNA sequencing and multi-level ecology analyses (α/β diversity, co-occurrence networks, and community assembly models). The results revealed that CBC increased bacterial richness and phylogenetic breadth relative to the unfertilized cultivated control, whereas OF showed dose-dependent declines in richness and CF maintained relatively stable richness with slight reductions in evenness at higher doses. Phylum-level composition shifted strongly with fertilizer identity: Bacillota decreased, whereas Pseudomonadota and Acidobacteriota increased under fertilization, with the largest compositional changes under CBC. CBC strengthened nutrient–enzyme–microbe coupling and generated increasingly complex, highly connected, and robust co-occurrence networks along the dose gradient, outperforming high-dose OF in network complexity and robustness, while OF maintained higher modularity. Null-model partitions (βNTI/RC_bray, NST, NCM, iCAMP) indicated that stochastic processes dominated community assembly across treatments; along the CBC gradient, dispersal limitation decreased from CBC1 to CBC2 and drift remained dominant, indicating increasing stochastic stabilization at moderate–high doses. Together, CBC promoted microbiome recovery and ecological resilience and represents a promising amendment for soil health.

## 1. Introduction

To meet the growing global demand for food driven by population expansion, agricultural systems have become increasingly reliant on conventional chemical fertilizers to boost crop yields and maximize output [1]. Since the Green Revolution, the use of chemical fertilizers—particularly nitrogen (N), phosphorus (P), and potassium (K)—has expanded rapidly worldwide [2]. However, issues such as the limited variety of fertilizers, high nutrient concentrations, and extensive yet imprecise application methods have become increasingly prominent, evolving into a trend of excessive and poorly regulated use [3]. While these fertilizers contribute to short-term yield improvements, they also cause serious environmental problems [4]. These include nitrogen leaching leading to eutrophication of water bodies, elevated nitrate levels in groundwater, increased greenhouse gas emissions, soil acidification, and heavy metal accumulation [5,6]. Traditional fertilizers often lack environmental compatibility and fail to establish a harmonious relationship with soil ecosystems, thereby accelerating agricultural ecological degradation [7]. Therefore, rather than merely focusing on increasing fertilizer quantities, greater attention should be given to optimizing and customized fertilizer types [8]. Promoting the research and adoption of environmentally friendly alternatives—such as slow-release fertilizers, water-soluble fertilizers, organic fertilizers, and biofertilizers—has become a crucial direction for achieving green and sustainable agricultural development worldwide [9,10].

Castor (*Ricinus communis* L.) is an important oilseed crop, widely cultivated due to its high adaptability to arid and nutrient-poor environments, as well as its strong biomass productivity [11]. Castor seeds are rich in castor oil, which possesses excellent thermal stability, lubricity, and viscosity properties, making it an ideal raw material for the production of high-performance lubricants [12]. In particular, castor oil is extensively used in the aerospace industry for the formulation of high-temperature lubricating oils [13]. After the extraction of castor oil, the residual castor cake meal is a major by-product and has been increasingly explored as an agricultural resource. For example, castor bean cake as an agricultural supplement [14], also can be used for the control of gastrointestinal parasites in pasture-finished sheep [15]. Moreover, due to its rich content of crude protein, organic matter, and essential plant nutrients such as nitrogen, phosphorus, and potassium, castor bean cake has been widely developed and utilized as an organic fertilizer for soil improvement, mitigates saline stress in basil plants [16], improves yield and quality in organic farming systems [17].

Soil microbial communities are a critical component of agricultural ecosystems, playing a central role in maintaining soil health, driving nutrient cycling, and regulating plant growth [18]. Beneficial microorganisms, such as nitrogen-fixing bacteria, phosphate-solubilizing bacteria, and root growth-promoting fungi, contribute to enhanced nutrient acquisition, stress resistance and overall crop performance [19]. However, despite these benefits, microbial communities are highly sensitive to environmental changes [20], and their structure can be influenced by various agricultural practices [21]. One of the most significant factors affecting microbial community structure is the use of fertilizers [22]. Fertilization practices, especially the application of synthetic fertilizers, can initially promote microbial growth by providing essential nutrients, which stimulate microbial activity and enhance nutrient cycling [23]. However, prolonged or excessive use of chemical fertilizers can disrupt the natural microbial balance, negatively affecting microbial diversity and altering community assembly patterns [24]. Understanding how microbial communities assemble and respond to fertilization is crucial for developing ecologically sustainable farming practices that maintain or enhance soil health over the long term.

Although castor bean cake shows considerable potential as a fertilizer in improving crop yield and quality, current research remains limited. Existing studies have indicated that castor cake can enhance peanut yield and quality by improving soil physicochemical properties and increasing soil enzyme activity [17]. However, systematic research on how castor bean cake affects the assembly of soil microbial communities and their ecological functions remains scarce. Here, high-throughput sequencing was employed to systematically investigate the effects of castor bean cake fertilizer application on soil microbial community structure. The results showed that castor cake significantly altered the compositional characteristics of the soil microbial community. Following its application, the complexity and stability of the microbial community network were enhanced to some extent, suggesting that castor cake may positively regulate microbial assembly processes while providing nutrients. This study elucidates the response mechanisms of microbial community structure under different fertilization regimes and provides theoretical support for the scientific application of castor bean cake and the assessment of its ecological effects.

## 2. Materials and Methods

### 2.1. Soil Collection and Treatments

The field experiment was carried out between 2022 and 2024 at the Institute of Agriculture and Animal Husbandry of Tongliao city, Inner Mongolia Autonomous Region, China. The site has a temperate continental climate (mean elevation 650 m; mean annual temperature 8.2 °C; frost-free period ~140 d) and mean annual precipitation of ~395 mm, most of which falls in July–August. The soil is classified as sandy soil and the region is typical semi-arid [17].

This experiment was a two-year field trial. In 2022, spinach was sown on 20 May and harvested on 28 September without fertilization to establish a cultivated, unfertilized reference. In 2023, spinach was sown on 23 May and harvested on 2 October under different fertilization regimes. A randomized complete block design was used, with five replicates per treatment. Each plot measured 1 m × 5 m with 50 cm row spacing and 10 cm hill spacing; two seeds were sown per hill, giving a planting density of approximately 13,300 hills per 667 m^2^. A 1 m buffer strip separated plots. Field management followed local agronomic practice. All fertilizers were applied once as a basal dose before sowing; no topdressing was used.

Three fertilizer treatments were tested alongside controls: castor bean cake, cow manure fertilizer and chemical fertilizer. The castor bean cake was supplied by the Key Laboratory of Castor Breeding of the State Ethnic Affairs Commission (Tongliao, Inner Mongolia, China) and was characterized by pH 6.20, organic C 69.0 g/kg, total N 3.0 g/kg, total P 1.4 g/kg, and total K 3.31 g/kg; available nutrients: 0.25 mg/kg alkali-hydrolyzed N, 2.70 mg/kg available P, and 9.40 mg/kg available K [17]. The cow manure fertilizer consisted of fermented cattle dung provided by the Tongliao Institute of Agriculture and Animal Husbandry (Tongliao, Inner Mongolia, China): pH 6.5 to 8.5, 14.5% organic matter, 0.20–0.45% total N, 0.15–0.25% total P, and 0.10–0.15% total K. The chemical fertilizer was a compound fertilizer (N:P_2_O_5_:K_2_O = 15:15:15, total nutrients ≥ 45%) obtained from Stanley Agricultural Group Co., Ltd. (Shandong, China). All fertilizers were oven-dried, ground, and sieved prior to application; the castor bean cake was additionally autoclaved to eliminate active microbial interference. The experiment included the following treatments: a control group (CK0, original, unfarmed soil), CK1 (soil after one spinach cycle without fertilization), and four fertilization treatments applied to CK1 soil, including castor bean cake biofertilizer (CBC) at 7500 kg/ha (CBC1), 15,000 kg/ha (CBC2), and 22,500 kg/ha (CBC3); chemical fertilizer (CF) at 225 kg/ha (CF1), 300 kg/ha (CF2), and 375 kg/ha (CF3); and organic fertilizer (OF) at 7500 kg/ha (OF1), 15,000 kg/ha (OF2), and 22,500 kg/ha (OF3). The fertilization rates were selected based on commonly recommended fertilization rates from our previous studies [17]. All the fertilizers were input as basal fertilizers at one time before planting, and no topdressing was applied. Each treatment was replicated five times.

In each plot, soils from the 0–20 cm layer were collected using a five-point sampling strategy (one sample from the center and four from the plot corners). The five cores were pooled and homogenized to generate one composite sample per plot. Composite soils were collected 30 days after fertilization for subsequent physicochemical measurements and microbial community analysis. One portion of each soil sample was immediately frozen at −80 °C for microbial DNA extraction and 16S rRNA gene high-throughput sequencing, and the other portion was used for soil physicochemical property and enzyme activity assays. Soil physicochemical parameters and enzyme activities were determined using commercial assay kits according to the manufacturer’s instructions (Sangon Biotech Co., Ltd., Shanghai, China). All treatments were performed with five biological replicates. The complete datasets are reported in a separate manuscript currently under review and were used here as predictors.

### 2.2. DNA Extraction, Library Construction, and Sequencing

A total of 55 soil samples were collected for the analysis of soil microbial community structure. Genomic DNA was extracted from approximately 0.1 g of soil per sample using the PowerSoil DNA Isolation Kit (QIAGEN, Hilden, Germany), according to the manufacturer’s instructions. The extracted DNA concentration and purity were determined using NanoDrop 2000 (Thermo Scientific, Wilmington, DE, USA). The V3–V4 region of the bacterial 16S rRNA gene was amplified using primers 338F (5′-ACTCCTACGGGAGGCAGCAG-3′) and 806R (5′-GGACTACHVGGGTWTCTAAT-3′), both containing Illumina adapter sequences. PCR products were purified using 2% agarose gel electrophoresis and quantified with a Qubit 4.0 fluorometer (Thermo Scientific, Wilmington, DE, USA). Library construction was performed using the NEXTFLEX Rapid DNA-Seq kit (Bioo Scientific, Austin, TX, USA). All samples were sequenced on the Illumina MiSeq platform (paired-end, 2 × 300 bp) by Majorbio Biotech Co., Ltd. (Shanghai, China). Illumina MiSeq platform was used to sequence the 16S rRNA amplicons. Sequencing data were processed using the appropriate bioinformatics pipelines to generate high-quality reads for downstream analysis.

### 2.3. Statistical Analysis

Raw reads were demultiplexed, trimmed and quality-filtered (fastp v0.19.6) and paired-end reads were merged (FLASH v1.2.11). High-quality reads were denoised with the DADA2 plugin in QIIME2 (2020.2) to infer amplicon sequence variants (ASVs) and remove chimeras. For downstream α- and β-diversity analyses, the feature table was rarefied to 26,187 reads per sample. Taxonomy was assigned to ASVs using QIIME2’s naïve Bayes classifier trained on the SILVA 16S rRNA database (v138). In this study, the classification of bacterial phyla follows the most recent phylogenetic taxonomy based on the International Code of Nomenclature of Prokaryotes (ICNP). Alpha diversity indices included ACE, Shannon, Chao1, and phylogenetic diversity (PD), Simpson and Pielou, while beta diversity was assessed using Bray–Curtis dissimilarity and visualized via principal coordinate analysis (PCoA). Community differences were further validated using non-parametric tests (Adonis, ANOSIM, and MRPP) [25]. Redundancy analysis (RDA) was used to explore relationships between soil physicochemical properties and microbial community structure, while mantel test was employed to identify which key environmental factors influenced soil properties and microbial diversity. The variation partitioning approach (VPA) was used to evaluate the relative important of fertilization method and environmental factors on bacterial community using “vegan” package. Random forest analysis was used to identify key soil properties and enzyme activities associated with microbial community structure. Linear regression was employed to assess the influence of environmental factors on community composition differences.

### 2.4. Co-Occurrence Network Analysis and Community Assembly Mechanisms

To further investigate synergistic or antagonistic interactions among microbial taxa under different treatments, co-occurrence networks were constructed using the pMENA (molecular ecological network analysis) pipeline [26]. Only ASVs present in more than 50% samples were retained to reduce background noise. ASV abundances were normalized to relative values, and key topological parameters of the networks—including degree, clustering coefficient, betweenness centrality, and modularity—were calculated. Networks were visualized using Gephi software (version 0.9.3), and keystone nodes and modules were identified based on centrality metrics [27]. Network robustness and vulnerability were assessed using algorithms described in the study by Yuan et al. [28]. Additionally, *Zi*-*Pi* graph analysis was used to identify keystone taxa within the networks. Based on the within-module connectivity (*Zi*) and among-module connectivity (*Pi*) of each node, key microbial taxa were categorized into four types: module hubs (*Zi* < 2.5 and *Pi* > 0.62), network hubs (*Zi* > 2.5 and *Pi* > 0.62), connectors (*Zi* > 2.5 and *Pi* < 0.62), and peripherals (*Zi* < 2.5 and *Pi* < 0.62).

To explore community assembly mechanisms, four ecological models were integrated: (1) The normalized stochasticity ratio (NST) was used to determine the dominant process—NST > 50% indicated stochastic assembly, while NST < 50% indicated deterministic processes [29]. (2) The neutral community model (NCM) was used to test for neutral dynamics, where parameter Nm reflects microbial dispersal ability, and R^2^ indicates model fit [30]. (3) The β-nearest taxon index (β-NTI) assessed phylogenetic community turnover—|β-NTI| > 2 indicated deterministic processes, while |β-NTI| < 2 suggested stochasticity [31]. (4) The Raup–Crick metric based on Bray–Curtis dissimilarity (RCbray) was used to distinguish types of stochastic processes: RC > +0.95 indicated dispersal limitation, RC < −0.95 indicated homogenizing dispersal, and values in between indicated drift [32].

Additionally, the iCAMP (integrated community assembly model by phylogenetic-bin-based null model) was introduced to quantify the relative contributions of deterministic and stochastic processes at the phylogenetic-bin level. The iCAMP model classified assembly mechanisms based on βNRI and RC metrics, identifying deterministic processes [heterogeneous selection (HeS), homogeneous selection (HoS)] and stochastic processes [drift (DR), dispersal limitation (DL), and homogenizing dispersal (HD)] [33].

### 2.5. Data Availability

All raw 16S rRNA bacterial sequencing data have been deposited in the NCBI Short Read Archive (SRA) database (PRJNA1297351).

## 3. Results

### 3.1. Effects of Different Fertilization Treatments on Soil Bacterial Community Composition and Alpha Diversity

**Figure 1:**
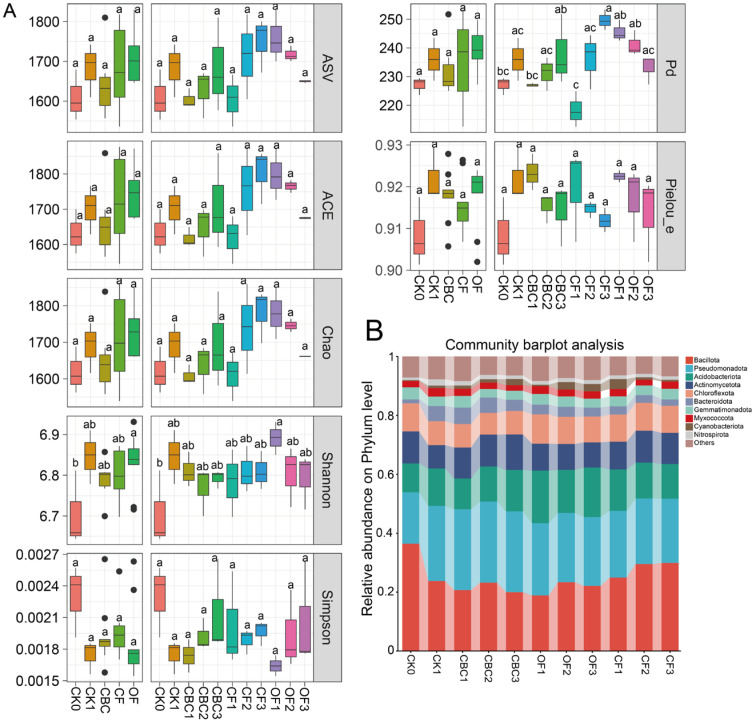
Dynamics of microbial community composition, α diversity, and β diversity under different fertilization treatments. (**A**) α-diversity indices across treatments. (**B**) Relative abundances of bacterial phyla. CK0, original soil; CK1, soil after one round of spinach cultivation without fertilization; CBC, castor bean cake biofertilizer at 7500 (CBC1), 15,000 (CBC2), and 22,500 kg/ha (CBC3); CF, chemical fertilizer at 225 (CF1), 300 (CF2), and 375 kg/ha (CF3); OF, organic fertilizer at 7500 (OF1), 15,000 (OF2), and 22,500 kg/ha (OF3). Different letters above boxplots indicate significant differences among treatments (*p* < 0.05). Black dots indicate outliers.

After removing non-target sequences including chloroplasts, mitochondria, archaea, and unassigned reads, a total of 3,337,504 high-quality bacterial sequences were obtained from 55 soil samples, with an average of 60,681 reads per sample (Table S1). Overall, 90,479 bacterial ASVs were identified across all samples (Figure 1A). Rarefaction curves approached saturation and Good’s coverage averaged 97.9%, consistent with a high sequencing depth across samples (Figure S1).

Bacterial richness and phylogenetic breadth were higher in CK1 than in the original uncultivated soil (CK0). Specifically, CK1 showed higher ASV richness, ACE, Chao1 and phylogenetic diversity (PD) than the original uncultivated soil (CK0), whereas community evenness and diversity indices (e.g., Simpson) changed only slightly (Figure 1A). Relative to CK1, bacterial diversity varied among fertilizer types and doses. CBC exhibited a clear dose-dependent enhancement in richness-related indices, including ASV richness, ACE, Chao1 and PD, with CBC3 showing the highest values among CBC treatments. In contrast, OF displayed an opposite dose-negative pattern, with OF1 generally showing the highest richness/PD and OF3 the lowest. CF did not show a consistent dose-dependent decline in richness-related indices; ASV richness, ACE, Chao1 and PD were largely stable or slightly higher at moderate–high CF doses. However, evenness (Pielou_e) tended to decrease slightly under higher CF inputs, whereas Shannon diversity varied modestly across doses and did not show a strong monotonic response for any fertilizer type (Figure 1A).

Across soils receiving different fertilizer types and doses, the top 10 bacterial phyla accounted for 91.61–94.06% of the total relative abundance (Figure 1B). Overall phylum-level profiles were broadly similar across treatments, but the relative abundance of dominant phyla varied significantly (Figure 1B). The most abundant phyla were Bacillota (18.81–36.39%), Pseudomonadota (17.44–27.49%), Acidobacteriota (9.81–17.81%), Actinomycetota (7.96–12.03%), and Chloroflexota (7.34–9.96%). Compared to CK, Bacillota decreased in all fertilized treatments, with particularly large reductions under OF and CBC treatments (35.60–47.91%). In contrast, Pseudomonadota increased significantly following fertilization, with the largest increase relative to CK observed under CBC (57.46%), followed by CK1 (35.95%), OF (35.27%), and CF (27.54%). Acidobacteriota also increased after fertilization.

### 3.2. Effects of Different Fertilization Treatments on Soil Bacterial Beta Diversity

**Figure 2:**
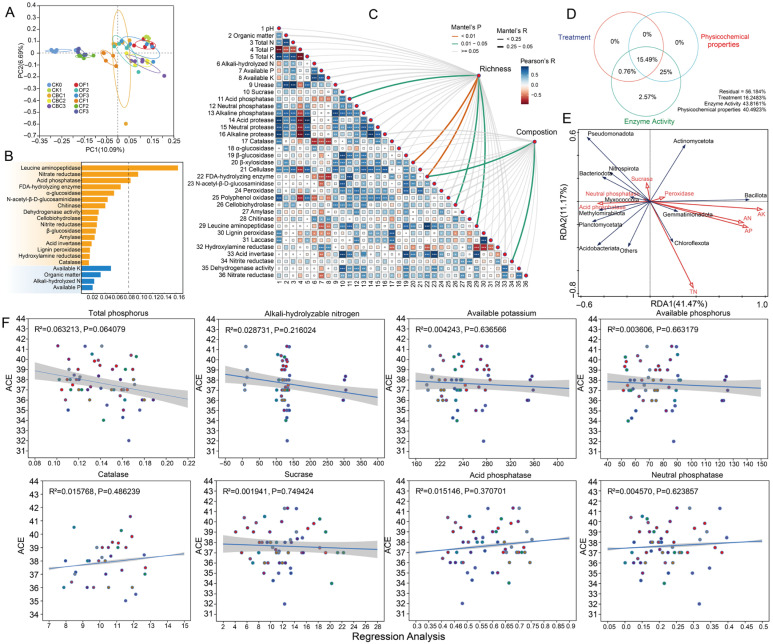
Effects of different fertilization treatments on soil bacterial β-diversity and environmental drivers. (**A**) PCoA based on Bray–Curtis dissimilarity at the ASV level. (**B**) Random-forest analysis identifying key factors shaping bacterial community composition. (**C**) Mantel test showing relationships between soil physicochemical properties and bacterial β-diversity. Square size is proportional to Pearson’s *r*. (**D**) Variation partitioning analysis (VPA) revealing the relative contributions of different treatments, soil characteristics, enzyme activity, and their interactions to the observed variation in microbial communities. (**E**) Redundancy analysis (RDA) showing how bacterial diversity is influenced by key soil physicochemical properties. (**F**) Linear relationships between bacterial richness (ACE index) and key soil properties and enzyme activities; solid lines are least-squares fits and shaded areas are 95% confidence intervals, with points representing samples from the different fertilization treatments. CK0, original soil; CK1, soil after one round of spinach cultivation without fertilization; CBC, castor bean cake biofertilizer at 7500 (CBC1), 15,000 (CBC2), and 22,500 kg/ha (CBC3); CF, chemical fertilizer at 225 (CF1), 300 (CF2), and 375 kg/ha (CF3); OF, organic fertilizer at 7500 (OF1), 15,000 (OF2), and 22,500 kg/ha (OF3). *** *p* < 0.001; ** *p* < 0.01; * *p* < 0.05.

Bray–Curtis PCoA at the ASV level showed that both the type and dose of fertilizer significantly reshaped soil bacterial β-diversity (*R* = 0.736, *p* = 0.001). As shown in Figure 2A, all three fertilization treatments were clearly separated from CK, indicating marked compositional shifts compared to the unfertilized control. The OF and CBC groups were closer to each other, with substantial overlap. Statistical tests further confirmed that fertilization treatment was a significant determinant of bacterial diversity (Table 1). Notably, OF1 partially overlapped with CK1, whereas OF2 was completely separated from CK1, indicating that bacterial community structure is influenced by both fertilizer type and application rate.

Random-forest modeling identified enzyme activities as the primary predictors of bacterial community structure, with leucine aminopeptidase (LAP) contributing the most, followed by nitrate reductase (NR), acid phosphatase (ACP), fluorescein diacetate hydrolase (FDAse), α-glucosidase (AG), N-acetyl-β-D-glucosaminidase (NAGase), and chitinase (CHI) (Figure 2B). Among the physicochemical variables, only available potassium (AK) showed comparatively high importance. Mantel tests indicated that community composition was more strongly correlated with environmental factors than richness (Figure 2C). Composition was significantly correlated with organic matter (OM), total phosphorus (TP), available potassium (AK) and multiple carbon–nitrogen–phosphorus-cycling enzymes [e.g., urease (URE), nitrate reductase/nitrite reductase (NR/NiR), dehydrogenase activity (DHA), leucine aminopeptidase (LAP), N-acetyl-β-D-glucosaminidase (NAGase), fluorescein diacetate hydrolase (FDAse), and acid/alkaline phosphatase (ACP/ALP)] (*p* < 0.05), whereas richness related mainly to soil organic carbon/organic matter (SOC/OM), total phosphorus (TP), available potassium (AK) and a few resource-acquisition enzymes (acid phosphatase (ACP), N-acetyl-β-D-glucosaminidase (NAGase), leucine aminopeptidase (LAP)).

The Pearson correlation matrix (Figure 2C) further showed predominantly positive associations among C/N-degrading hydrolases and between phosphatases and available P. Variance partitioning (VPA, Figure 2D) showed that enzyme activity and soil physicochemical properties, together with their interaction, accounted for most of the explained variation (43.8161% and 40.4923%, respectively), suggesting that fertilization influences microbial communities indirectly by altering environmental conditions. Redundancy analysis (RDA, Figure 2E) further revealed that the availability of P, K, and NH_4_^+^ forms the primary gradient, and—together with key enzymes (notably peroxidase (POD))—reshapes phylum-level distributions. Nutrient availability was the main driver, with enzymatic processes providing secondary differentiation. Regression analyses (Figure 2F) showed weak linear relationships between ACE richness and the measured soil properties (TP, AN, AK, AP) and enzyme activities (CAT, sucrase, acid/neutral phosphatases) (*R^2^* = 0.002–0.063; all *p* > 0.05), with only TP exhibiting a marginal negative trend (*R^2^* = 0.063, *p* = 0.064). Therefore, within the observed ranges, richness responds little to any single factor and is likely governed by multivariate coupling and/or nonlinear controls. Overall, community composition is more sensitive to nutrient availability and enzymatic processes than richness.

### 3.3. Bacterial Co-Occurrence Network Dynamics Under Different Fertilizer Treatments

**Figure 3:**
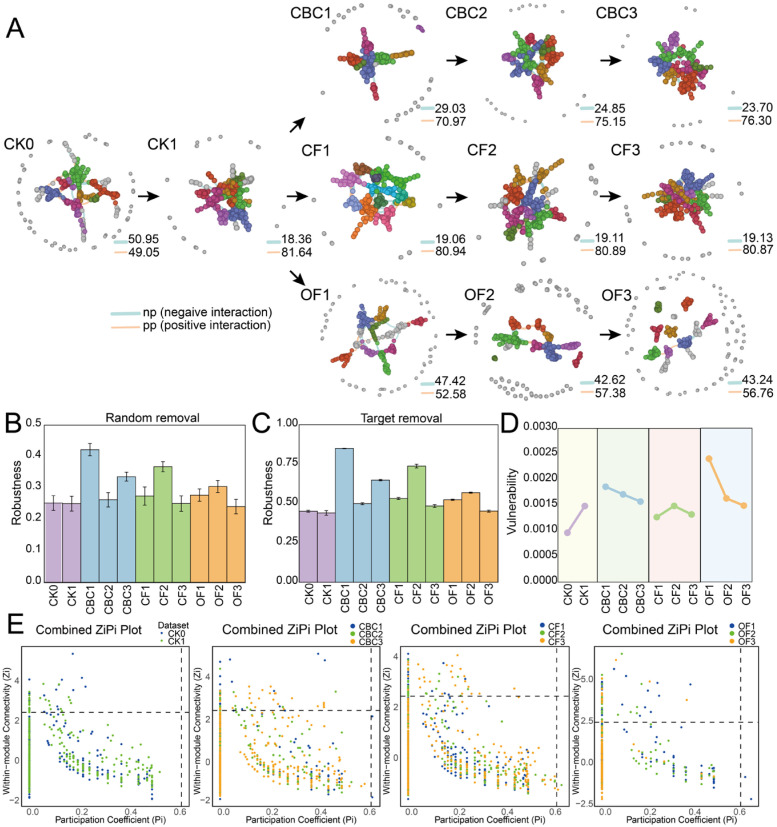
Co-occurrence networks of soil microbial communities based on Pearson correlations among ASVs. (**A**) Edges: orange, significant positive correlations; blue, significant negative correlations. Nodes represent ASVs and node colors indicate module membership. (**B**,**C**) Network robustness, quantified as the proportion of taxa remaining after random removal of 50% of nodes (**B**) or targeted removal of high-centrality nodes (**C**). (**D**) Network vulnerability, defined by the maximum node vulnerability within each network. (**E**) Keystone taxa inferred from the Zi–Pi plot; each filled circle denotes an ASV, with colors indicating fertilization treatments. CK0, original soil; CK1, soil after one round of spinach cultivation without fertilization; CBC, castor bean cake biofertilizer at 7500 (CBC1), 15,000 (CBC2), and 22,500 kg/ha (CBC3); CF, chemical fertilizer at 225 (CF1), 300 (CF2), and 375 kg/ha (CF3); OF, organic fertilizer at 7500 (OF1), 15,000 (OF2), and 22,500 kg/ha (OF3). pp: positive interaction; np: negative interaction.

We constructed eleven ASV-level co-occurrence networks (CK0, CK1, three CBC, three CF, three OF) to probe interaction patterns among soil bacteria. All empirical networks were non-random (*p* < 0.01) and exhibited hallmarks of complex systems—higher clustering and modularity than their randomized counterparts together with small-world/scale-free structure (Table 2; Figure 3). CBC showed a dose-positive trajectory: network size and connectivity increased from CBC1 to CBC3 (avgk 8.44 → 10.73), and average path length increased at CBC2 and then slightly decreased at CBC3. CF networks were consistently dense (avgk 9.41–10.06) with relatively high clustering (avgCC up to 0.394), pointing to a stable, well-connected configuration across doses, although CF3 showed the lowest clustering (avgCC 0.285). In contrast, OF showed a non-monotonic dose response, with network connectivity remaining relatively high at OF2 but dropping sharply at the highest dose; OF3 exhibited the sparsest topology (avgk 4.68 → 5.80 → 3.33), accompanied by very high modularity (0.865–0.897) and elevated betweenness centralization at OF1–OF2 (CB ≈ 0.20–0.21), suggesting that connectivity and clustering were generally higher in CBC and CF than in OF3. Robustness analyses confirmed these patterns. Targeted removal of high-degree nodes always reduced connectivity more than random removal. Across treatments, network robustness was generally associated with higher connectivity and clustering, but did not increase monotonically with dose. CBC1 and CBC3, together with CF2, retained connectivity longer under both random and targeted node removal, whereas the sparser networks (e.g., OF3 and CF3) degraded faster (Figure 3B,C; Table 2). *Zi–Pi* analysis identified treatment-specific keystone ASVs (module hubs/connectors) (Table S2). Positive correlations dominated all networks (Figure 3A), suggesting prevalent co-occurrence or cooperative niche use, while the high modularity across treatments indicated strong functional compartmentalization.

### 3.4. Assembly Mechanisms of Bacterial Communities Under Fertilization Regimes

**Figure 4:**
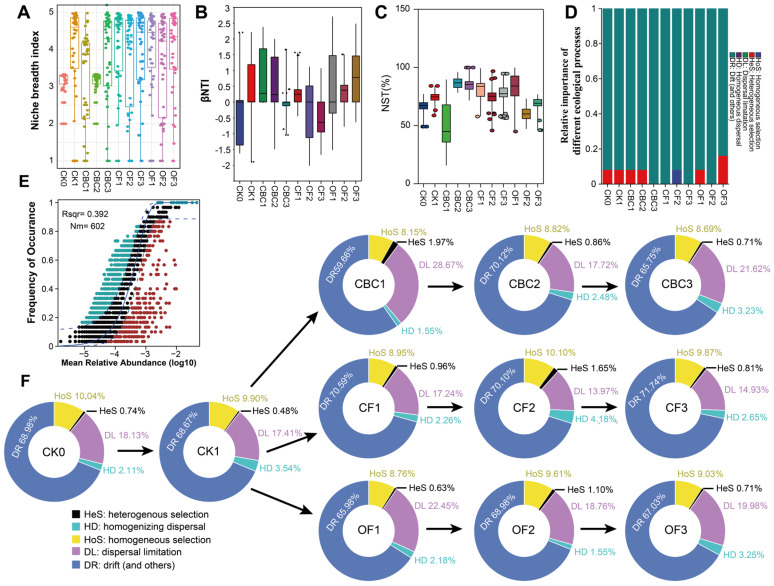
Evaluation of bacterial community assembly under different fertilization treatments. (**A**) Niche breadth. (**B**) Distribution of β–nearest taxon index (β-NTI) among samples. (**C**) Normalized stochasticity ratio (NST). (**D**) Relative importance of ecological processes from null-model partitioning: heterogeneous selection (HeS), homogeneous selection (HoS), dispersal limitation (DL), homogenizing dispersal (HD), and drift and others (DR). (**E**) Sloan’s neutral community model (NCM); blue solid line indicates the model fit and blue dashed lines the 95% confidence interval. (**F**) Relative importance of ecological processes at the phylogenetic-bin scale from iCAMP. CK0, original soil; CK1, soil after one round of spinach cultivation without fertilization; CBC, castor bean cake biofertilizer at 7500 (CBC1), 15,000 (CBC2), and 22,500 kg/ha (CBC3); CF, chemical fertilizer at 225 (CF1), 300 (CF2), and 375 kg/ha (CF3); OF, organic fertilizer at 7500 (OF1), 15,000 (OF2), and 22,500 kg/ha (OF3).

To link the diversity and network patterns above with the underlying assembly rules, we combined niche breadth, β-nearest taxon index (βNTI), normalized stochasticity ratio (NST), Sloan’s neutral community model (NCM), and the phylogenetic-bin–based null model iCAMP (Figure 4A–F). Firstly, the niche breadth index was used to assess the environmental tolerance and resource-use range of the bacterial community across treatments. Relative to CK0, CK1 showed a slight increase, whereas all fertilizer treatments broadened niches. CF and OF maintained consistently high niche breadth across doses, while CBC displayed a clear dose response: lower and more variable breadth at CBC1–CBC2, then an increase at CBC3 approaching the CF/OF levels (Figure 4A). Thus, fertilization—especially high-dose CBC—expanded the resource-use range and environmental tolerance of bacterial communities. We then used these null-model–based metrics to partition community turnover into components attributable to stochastic processes versus environmental selection. Because most samples had β-nearest taxon index (βNTI) values between −2 and +2, stochastic processes were identified as the primary drivers of assembly (Figure 4B). We further used NST as an index describing the balance between stochasticity and determinism in community assembly. The NST values were above 50% across treatments, indicating stochastic dominance of assembly. A clear dose effect appeared in CBC: NST increased from CBC1 to CBC3. CF maintained high NST across doses, whereas OF showed greater variability (Figure 4C, Table S3).

Weighted βNTI values were analyzed together with the Bray–Curtis–based Raup–Crick index (RC_bray) to partition community turnover among different assembly processes. When taxonomic information was not incorporated, drift (DR) emerged as the dominant process (βNTI < 2, Figure 4B), explaining >90% of community turnover, followed by heterogeneous selection and homogeneous selection (Figure 4D). We also applied Sloan’s neutral community model (NCM) to test whether ASV occurrence frequencies across samples were consistent with neutral expectations. The fitted model (R^2^ = 0.392, Nm = 602) showed that most taxa fell within the 95% confidence envelope (Figure 4E), which is in line with a predominant influence of stochastic, neutral-like processes on bacterial community assembly in these soils.

Finally, we applied iCAMP to infer assembly mechanisms under different fertilizers (Figure 4F). Across treatments, drift (DR) dominated (67–72%). Dispersal limitation (DL) was the main secondary process, highest at CBC1 (28.66%) and OF1 (22.44%), and lower in CF (about 14–18%). Along the CBC dose gradient, DL decreased from CBC1 to CBC2 and drift remained dominant, indicating increasing stochastic stabilization at moderate–high doses. CF maintained high DR and low DL across doses; only CF2 showed a small peak of homogenizing dispersal (4.17%). OF showed elevated DL at OF1 that decreased with dose with a concomitant rise in DR. Homogeneous selection (HoS) remained 8–10%, and heterogeneous selection (HeS) was negligible. Overall, fertilizer type and dose primarily tune the DR–DL balance rather than impose strong selection, consistent with the βNTI and NST results.

## 4. Discussion

Fertilization methods are a key factor influencing the structure and ecological functions of soil microbial communities [34]. However, a broader body of work shows that soil microbiomes are also shaped by management practices and environmental gradients, including land use, geography, and depth stratification [35,36]. These studies highlight that fertilization effects should be interpreted within a wider context of agronomic management and environmental heterogeneity. Our multi-dimensional analysis therefore focused on how castor bean cake, chemical fertilizers, and cattle manure, applied at different doses, modulate bacterial community composition, diversity, interaction networks, and assembly processes against this broader ecological background. The results demonstrated significant variations among fertilizer types in terms of ecological disturbance intensity, resilience, and regulatory mechanisms. Notably, the castor cake treatment exhibited strong ecological modulation potential and system restructuring capacity.

### 4.1. Castor Cake Application Promotes the Enrichment of Functional Bacterial Phyla and Diversity Recovery

Community composition is a sensitive indicator of fertilization effects [37]. Castor bean cake markedly reshaped the bacterial community, with fertilizer-specific and dose-dependent shifts at both taxonomic and diversity levels. At the phylum level, Bacillota decreased after fertilization, including all CBC treatments, whereas copiotrophic groups such as Pseudomonadota and Acidobacteriota increased, with the largest compositional turnover observed under CBC (Figure 1B). Although the response was not strictly dose-linear, Actinomycetota showed a slight enrichment tendency under CBC compared with the cultivated control and chemical fertilizer, remaining one of the major decomposer lineages. Actinomycetota are key degraders of complex organic carbon and are often linked to antibiotic biosynthesis and suppression of soil-borne pathogens [38,39]. Their maintenance or modest enrichment under CBC likely reflects the abundant polysaccharides, fatty acids, and protein-derived substrates released during castor-cake decomposition, which broaden available niches for organic-matter–specialized taxa [40,41].

At the diversity level, CBC promoted a dose-dependent recovery of richness and phylogenetic breadth. Richness-related indices (ASV richness, ACE, Chao1) and phylogenetic diversity increased from CBC1 to CBC3 (Figure 1A), indicating that higher CBC inputs broadened resource niches and supported more taxa. Shannon diversity varied modestly without a strong monotonic pattern, but high-dose CBC maintained or slightly improved overall diversity relative to CK1. In addition, CBC favored several plant-beneficial bacterial genera (e.g., *Pseudomonas* and *Bacillus*) [42], which are associated with rapid utilization of labile substrates and antagonism against soil-borne pathogens. Such CBC-induced shifts in community composition and diversity are consistent with broader observations that organic inputs and reduced chemical fertilization can enrich diverse and functionally important taxa in managed soils [35,36]. Overall, CBC provides readily decomposable organic carbon and nutrients, supports decomposer- and plant-beneficial groups, and enhances bacterial richness and phylogenetic breadth, highlighting its potential for improving soil microecological resilience.

### 4.2. Castor Cake Mediates Microbial Community Restructuring Through Nutrient and Enzyme System Modulation

Castor bean cake is a natural fertilizer rich in organic matter and diverse bioactive compounds [43]. In this study, it reshaped bacterial communities mainly through resource enrichment and enzyme-mediated substrate turnover, rather than through a single physicochemical driver. CBC inputs increased soil organic matter and total P, and simultaneously stimulated activities of multiple extracellular enzymes involved in C–N–P cycling. Such coupled changes indicate that CBC rapidly generates a substrate-rich microenvironment that promotes microbial metabolic activation and niche expansion, a pattern commonly reported for high-quality organic amendments [44,45]. In line with this mechanism, Mantel tests and ordination analyses showed that community composition was tightly linked to organic matter/soil organic carbon, total phosphorus, available potassium, and key C–N–P acquisition enzymes (e.g., urease, phosphatases, leucine aminopeptidase, N-acetyl-β-D-glucosaminidase, and fluorescein diacetate hydrolase; Figure 2B), suggesting that biochemical processes of decomposition and nutrient release are central to CBC-driven turnover.

Notably, random-forest modeling identified enzyme activities as the strongest predictors of community structure, whereas among soil properties only available potassium showed relatively high importance (Figure 2A). This supports the view that CBC regulates microbiomes indirectly by accelerating organic matter depolymerization and nutrient mineralization through enhanced enzyme systems, thereby favoring taxa capable of rapid substrate use and cross-feeding [46]. Compared with chemical fertilizer, which supplies readily available inorganic nutrients but little organic carbon, CBC is more effective at stimulating coordinated enzyme responses and sustaining resource heterogeneity, which helps maintain higher richness and broader phylogenetic breadth [47]. In contrast, manure-based organic fertilizer can produce weaker or more variable microbial regulation because its substrates are chemically heterogeneous and decompose more slowly, delaying enzyme–nutrient feedbacks [48]. Overall, CBC establishes a nutrient–enzyme–microbe feedback loop: high-quality organic substrates and phosphorus inputs elevate key enzyme activities, which in turn accelerate nutrient release and reinforce microbial succession toward a more functionally active and resilient community [49]. This mechanism explains why CBC produced stronger compositional reorganization and richer ecological linkages than CF and high-dose OF, highlighting its promise for sustainable soil improvement.

### 4.3. Effects of Different Fertilizers Application on Soil Microbial Ecological Network

Microbial taxa interact to form complex co-occurrence networks, and such analyses help infer potential ecological interactions relevant to ecosystem functioning [50,51]. Previous studies report that fertilization can alter nutrient dynamics, stimulate specific microbial taxa, and intensify interspecific interactions, thereby enhancing community stability and function [52]. In our study, fertilization clearly reshaped bacterial interaction patterns (Figure 3A; Table 2), but the direction and magnitude depended on fertilizer type and dose. Specifically, high-dose OF reduced average degree, clustering coefficient, and betweenness centralization, indicating weakened connectivity and lower network complexity. In contrast, CF and CBC increased average degree and degree centralization, suggesting strengthened connectivity and a greater role of hub taxa. These patterns agree with previous reports that organic amendments can enhance microbial interactions at moderate inputs but may destabilize networks when excessive recalcitrant substrates accumulate and overwhelm decomposition capacity [53,54].

Keystone taxa identified by Zi–Pi analysis (module hubs and connectors) are crucial for maintaining network integrity and functional flow [55]. Keystone ASVs differed among fertilizers: under CF, they were mainly affiliated with Pseudomonadota and Bacillota, consistent with copiotrophic traits and strong enzyme production that favor central positions in nutrient-enriched environments [56]. Along the CBC gradient, keystone ASVs were more frequently assigned to Chloroflexota and Acidobacteriota, with Pseudomonadota also involved, implying that CBC inputs promote cross-phylum cooperation and within-module organization under organic-carbon enrichment [57]. In OF treatments, keystones belonged to Nitrospirota, Cyanobacteriota, Planctomycetota, and Gemmatimonadota, indicating functional channels linked to nitrogen cycling, phototrophic niches, or adaptation to comparatively resource-limited conditions. Across all networks, positive correlations dominated, suggesting prevalent cooperative or shared-niche associations, while negative links likely reflect competition that can prevent overdominance and contribute to stability [58,59]. Overall, CBC enhanced network connectivity and robustness while maintaining high modular organization, indicating stronger microbial interaction capacity than high-dose OF and generally comparable or stronger architecture than CF. This highlights CBC as an efficient organic amendment for fostering resilient soil microbial networks in sustainable agriculture.

### 4.4. Stochastic and Deterministic Processes Structure Bacterial Community Assembly

Soil microbial communities are shaped jointly by deterministic and stochastic processes, and their balance governs community responses to environmental variation and ecological stability [60,61]. Integrating niche breadth, βNTI, NST, and iCAMP (Figure 4), we found that assembly was predominantly stochastic across treatments, driven largely by random encounters and colonization [62]. Stochastic-dominated communities often show higher robustness within complex networks [63], consistent with previous observations in soil microbiomes [64]. Under CBC, low doses showed relatively stronger environmental filtering, whereas moderate–high doses shifted rapidly toward stochastic dominance, suggesting high adaptive capacity; fertilization may also promote microbial aggregation and cooperation, as reported previously [65].

To further characterize assembly processes, we applied the scheme of Ning et al. [33], which splits community turnover into five categories: DL, HeS, HD, HoS and DR, and used this classification to evaluate how strongly each process contributed. Drift, dispersal limitation, and homogeneous selection explained most community turnover (Figure 4F), likely reflecting nutrient-enriched conditions that enhance demographic variability and stochasticity [66,67]. CBC contains high nitrogen (7.54%), comparable to cottonseed meal (8.21%) and higher than many other amendments [68]. Under nitrogen enrichment, key nutrient-turnover guilds are less constrained by environmental filtering and more influenced by birth–death–dispersal events [69,70], which can broaden niche breadth under high N availability [71], consistent with our results (Figure 4A). Mechanistically, HoS represents the main deterministic pathway and DR the dominant stochastic pathway [72]. When dispersal is limited, drift becomes more influential, producing abundance fluctuations [73,74]. In our data, CF showed a relatively higher DR fraction, whereas CBC and OF were lower, indicating a more balanced drift–dispersal regime under organic inputs [75]. Along the CBC gradient, dispersal limitation decreased from CBC1 to CBC2 and drift remained dominant, indicating increasing stochastic stabilization at moderate–high doses (Figure 4F). Dispersal signals were strongest under CBC, intermediate under OF, and weakest under CF, reinforcing CBC’s advantage.

Collectively, these findings highlight the primacy of stochastic processes in structuring fertilized soil microbiomes. CBC not only promotes drift-modulated stochastic assembly at moderate–high doses but also enhances cross-phylum cooperation, supporting robust community reassembly. Lima et al. reported that CBC mineralizes rapidly and generally does not require composting [76], making it recyclable and aligned with green-agriculture goals. However, high nitrogen inputs can become phytotoxic at elevated doses, and CBC may contain ricinine and ricin-related compounds with ecological risks [77]. Future work should define safe application windows and mitigation practices, including multi-soil field trials and monitoring of leachates and edible tissues.

## 5. Conclusions

This study shows that castor bean cake (CBC) drives distinct, dose-dependent changes in soil bacterial communities compared with chemical fertilizer (CF) and manure-based organic fertilizer (OF). CBC increased bacterial richness and phylogenetic breadth along the dose gradient, whereas OF showed a dose-negative trend and CF maintained richness but slightly reduced evenness at higher doses. Fertilization shifted phylum composition, with Bacillota decreasing and Pseudomonadota and Acidobacteriota increasing; CBC caused the strongest compositional turnover and slightly favored organic-matter decomposers such as Actinomycetota. CBC-enhanced nutrients and C–N–P cycling enzymes were closely linked to these shifts and to progressively more connected and robust co-occurrence networks, outperforming high-dose OF in network stability. Null-model analyses indicated stochastic assembly dominance; along the CBC gradient, dispersal limitation decreased from CBC1 to CBC2 while drift remained dominant, indicating increasing stochastic stabilization at moderate–high CBC doses. Overall, CBC is a promising organic amendment for improving soil microbial diversity and resilience, provided safe application rates are ensured.

## Figures and Tables

**Table 1:** Results of multivariate analyses (Adonis, ANOSIM and MRPP) evaluating differences in soil bacterial community composition among fertilization treatments.

	Adonis		ANOSIM		MRPP	
	*F*	*p*	*R*	*p*	*δ*	*p*
Samples	9.1505	0.001	0.6081	0.001	0.3936	0.001

**Table 2:** Summary of network-topology metrics for bacterial co-occurrence networks under different fertilization treatments and for their corresponding random networks.

Network Name	Topological Properties	CK0	CK1	CBC1	CBC2	CBC3	CF1	CF2	CF3	OF1	OF2	OF3
Empirical	Similarity threshold	0.97	0.97	0.99	0.97	0.97	0.97	0.97	0.97	0.97	0.97	0.98
	Total nodes	699	683	529	638	664	705	679	749	629	610	497
	Total links	2836	3157	2232	2929	3561	3546	3239	3523	1472	1769	828
	Average degree (avgk)	8.114	9.245	8.439	9.182	10.726	10.060	9.541	9.407	4.680	5.800	3.332
	Centralization of degree (CD)	0.049	0.054	0.134	0.052	0.081	0.048	0.049	0.050	0.050	0.076	0.056
	Average path distance (GD)	6.696	5.932	4.170	5.747	5.598	6.297	6.109	5.752	7.060	5.620	5.496
	Average clustering coefficient (avgCC)	0.443	0.352	0.330	0.312	0.351	0.394	0.326	0.285	0.370	0.404	0.377
	Centralization of betweenness (CB)	0.218	0.106	0.233	0.087	0.140	0.106	0.101	0.105	0.212	0.201	0.100
	Modularity	0.829	0.799	0.692	0.795	0.730	0.810	0.772	0.763	0.865	0.822	0.897
Random networks	Modularity	0.308 ± 0.004	0.286 ± 0.004	0.298 ± 0.004	0.288 ± 0.004	0.258 ± 0.004	0.271 ± 0.003	0.281 ± 0.004	0.285 ± 0.004	0.456 ± 0.005	0.386 ± 0.004	0.583 ± 0.006
	Average path distance (GD)	3.294 ± 0.019	3.142 ± 0.012	3.090 ± 0.020	3.140 ± 0.013	2.970 ± 0.011	3.065 ± 0.013	3.121 ± 0.012	3.157 ± 0.011	3.919 ± 0.029	3.572 ± 0.024	4.607 ± 0.058
	Average clustering coefficient (avgCC)	0.032 ± 0.003	0.029 ± 0.003	0.049 ± 0.004	0.025 ± 0.002	0.043 ± 0.003	0.033 ± 0.002	0.029 ± 0.003	0.027 ± 0.002	0.020 ± 0.003	0.030 ± 0.004	0.013 ± 0.004

## Data Availability

All raw 16S rRNA bacterial sequencing data have been deposited in the NCBI Short Read Archive (SRA) database at https://www.ncbi.nlm.nih.gov/bioproject/?term=PRJNA1297351, reference number PRJNA1297351, accessed on 28 July 2025.

## Supplementary Materials

The following supporting information can be downloaded at: https://www.mdpi.com/article/10.3390/microorganisms13122841/s1, Figure S1: Good’s coverage curves of soil bacterial communities across all treatments; Table S1: Sequencing data summary for soil bacterial communities across all samples; Table S2: The keystone ASV in each co-occurrence network; Table S3: The normalized stochasticity ratio (NST) in different bacterial communities.
