# Supplementary figures and images for: Ecological Modulation of Soil Microbial Communities by Fertilization Regimes: Insights from Castor Bean Cake, Chemical Fertilizers, and Organic Fertilizer
Source: Microorganisms. 2025 Dec 14;13(12):2841. doi: 10.3390/microorganisms13122841 (PMC12735973; doi:10.3390/microorganisms13122841)

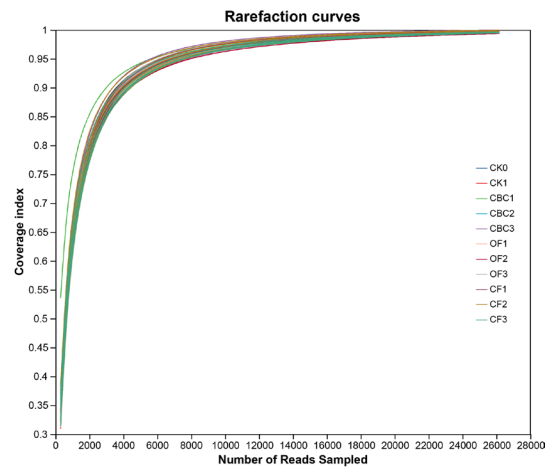

Fig. S1 Good's coverage curves of soil bacterial communities across all treatments

Supplement: Supplementary file 1 [file microorganisms-13-02841-s001.zip › Supplementary Figure.pdf]
